# Incidence of diabetes mellitus-related comorbidities among patients attending two major HIV clinics in Botswana: a 12-year retrospective cohort study

**DOI:** 10.1186/s13104-018-3144-9

**Published:** 2018-02-01

**Authors:** Goabaone Rankgoane-Pono, Jose Gaby Tshikuka, Mgaywa Gilbert Mjungu Damas Magafu, Tiny Masupe, Mooketsi Molefi, Shimeles Genna Hamda, Vincent Setlhare, Roy Tapera, Bontle Mbongwe

**Affiliations:** 1 Department of Family Medicine and Public Health, Faculty of Medicine, University of Botswana, Gaborone, Botswana; 2 Department of Health Sciences, National Pedagogic University, Kinshasa I, Democratic Republic of the Congo; 3 Department of Global Health, University of Washington, Seattle, USA; 4 School of Public Health, Faculty of Health Sciences, University of Botswana, Private Bag 0022, Gaborone, Botswana

**Keywords:** Incidence, Diabetes-related comorbidities, Combination antiretroviral therapy, PLHIV, Botswana

## Abstract

**Objectives:**

Exposure to combination antiretroviral therapy (cART) is associated with the development of diabetes mellitus related comorbidities (DRCs). This study aims to: (i) estimate the incidence of DRCs among cART recipients, (ii) assess the time-to-event (development of DRC) and, (iii) compare survival function between recipients on first-line regimen and those on second-, third-line cART regimen.

**Results:**

The incidence of DRCs was 26.8/1000 person-years, with total time of exposure of 3316 person-years. The average time to event for all the three regimens was 11.72 ± 0.20 years. The first-line cART regimen had a shorter mean ± SE of 10.59 ± 0.26 years to the event compared to 12.69 ± 0.24 years for the second-, third-line cART regimen. Recipients on the first-line had a shorter survival than recipients on second-, third-line cART (Log-rank X^2^ = 8.98, p < 0.003). Data from this study showed that the risk of developing DRCs per year of exposure was significantly greater for patients on first-line compared to those who were on second-, third-line regimen; which, suggests that monitoring of cART long-term side effects and regular reviewing of cART regimens is important. Meticulous selection of drug combinations is a key to improving recipients’ survival.

**Electronic supplementary material:**

The online version of this article (10.1186/s13104-018-3144-9) contains supplementary material, which is available to authorized users.

## Introduction

HIV as a condition along with treatment with combination antiretroviral therapy (cART) are both known risk factors for Type 2 diabetes [1–3]. Studies have shown that after a few years of follow up, a significant number of patients on cART develop Type 2 diabetes or related comorbidities compared to HIV-negative controls [3]. Therefore, countries adversely affected by the HIV epidemic where long-term cART has been available, like Botswana, have the potential for a dual HIV/AIDS-diabetes related comorbidities (DRC) epidemic [2, 4–6].

Different mechanisms have been suggested to explain this occurrence; many of which are drug class specific. Nucleoside reverse transcriptase inhibitors (NRTIs) such as stavudine, zidovudine, lamivudine and didanosine are reported to be associated with DRCs through mitochondrial toxicity, lipodystrophy or pancreatitis [1, 7]. Non-nucleoside reverse transcriptase inhibitors (NNRTIs), like efavirenz and nevirapine, which are used in first-line regimens for HIV treatment in most of the sub-Saharan African region are believed to be rarely associated with DRCs [8]. This is in spite of the fact that drugs such as nevirapine have been linked to an increased low-density lipoprotein (LDL) [9]. On the other hand, long use of efavirenz has been shown to increase total blood cholesterol and triglycerides [10]. Protease inhibitors (PIs), e.g. indinavir, have all been implicated in causing abnormal glucose levels among PLHIV [1, 11]. DRCs seem to be a function of both cART use and its duration. However, this does not exclude the presence of other factors such as lack of knowledge about cART adverse effects [7].

A preliminary discussion with health care workers at HIV clinics in Gaborone, and surrounding areas in Botswana, revealed a lack of knowledge about the high occurrence of DRCs among cART recipients and which cART provided the longest survival to the occurrence of DRCs. Yet, this information is crucial for planning interventions that minimise morbidity and mortality among PLHIV in Botswana and other countries responding to the HIV/AIDS epidemic. There is need to describe and determine the public health impact of DRCs among cART recipients in order to develop strategies for prevention and effective long-term disease management. Therefore it is critical that the number of new cases of DRCs occurring during a time period among recipients on specific cART regimen and exposure time duration be known and be monitored. We aim in this study to: (i) estimate the incidence of DRCs among cART recipients, (ii) assess the time-to-event and, (iii) investigate whether the survival function is the same between recipients on first-line cART regimen and those on second-, third-line cART regimen.

## Main text

### Operational definitions

In this study diabetes-related comorbidity (DRCs) was any comorbidity associated with type 2 diabetes as defined in ICD-10-CM Codebook Index [12]. Combination antiretroviral therapy regimens were categorized as defined by the Botswana National HIV & AIDS Treatment Guidelines [13] and the Handbook of the Botswana Integrated HIV Clinical Care Guidelines [14] in use between 2002 and 2015. Details on what the first and second line regimens were made up of are given in the Additional file 1: Appendix 1.

### Study design and site

This study was a 12-year retrospective cohort analysis of cART recipients at two major HIV clinics in Gaborone, Botswana. Gaborone has 230,000 inhabitants as of 2011 [14]. HIV/AIDS period prevalence (2008–2013) was estimated to be 19% among persons aged 18 months and above [15]. Two health clinics were selected as study sites, Princess Marina Hospital (PMH) HIV clinic and Bontleng HIV clinic based on their capacity to provide care to a large number of HIV patients and the high quality of their record keeping. The exposure variable for this study was “use of cART” and the outcome variable was the “occurrence of DRCs” as diagnosed by a treating physician.

### Sample size

The sample size was determined using a sampling error of 0.05 and a beta level of 0.20 [16]. The proportion of baseline DRC among recipients of cART was 17.6% [4] and the expected magnitude of association between cART and DRC was set at 1.9 odds ratio. This led to an estimated sample size of 483 which was increased by 11.9% or the proportion of PLHIV with DRCs [17] before the study begins and thus bringing the sample to 540 participants.

### Data collection

Client medical record numbers from both clinics were used to form the sampling frame. The computer table of random numbers was used to select 540 patients. Their medical records were used as the source of the data. Only HIV positive patients were included in the study. The following data points were collected from patient files: age, gender, date of enrolment into the programme, date of cART initiation, weight (in kilogrammes) at cART initiation (Weight-1) and at the time of data collection (Weight-2), height (in centimetres) when entering the programme, CD4 cell count at cART initiation (CD4-1), CD4 cell count at the time of data collection (CD4-2), cART regimen received and whether adherence to the treatment had been maintained or interrupted. Information on DRCs as well as the date of diagnosis was also collected. Two groups of patients were identified as follows: (i) patients who received first-line cART and (ii) those who received second-line/third-line cART. They were followed up from 2002 to 2015. The follow up endpoint was when a diagnosis of DRCs was made or the end of the study. We excluded patients who had DRCs at entry into the treatment programme, pregnant women, patients initiated on cART after the year 2012 (allowing at least 3 years of follow-up for patients initiated in 2012), patients aged less than 18 years and those with discrepancies in data from records within the same clinic.

### Data analysis

IBM SPSS version 21 (Chicago, IL) was used for analysis. Different types of DRCs namely high blood pressure, hypertension, renal failure, overweight, cardiovascular conditions and lipodystrophy were identified. The frequency distribution (%) of cART recipients with or without DRCs and the distribution of DRCs among patients were computed. The proportion of patients who were on first-line, second-line or third-line cART regimens was also computed. Patient CD4 cell count at enrolment (CD4-1) and at the onset of the DRCs (CD4-2) were collected and the mean [(standard error of the mean (SEM)] were estimated. Patients with CD4-1 and CD4-2 ≤ 200 cells/mm^3^ and those with CD4-1 and CD4-2 ≥ 350 cells/mm^3^ were identified and compared. Comparisons were made as paired samples using McNemar’s test.

In this study, incidence rate was considered a function of the duration of exposure to specific cART. To compute the incidence of DRCs among recipients, the rate of new onset DRCs was computed as the number of new cases divided by the total person-years of follow-up (PY). The PY estimated the actual time-at-risk in years that all participants contributed to the study. Since the event or outcome was DRCs, survival was calculated as the time elapsed from the date of cART initiation to the date of the development of the first DRC or the end of the study. Cases of loss to follow up or death were censored at the last time they were seen (left censored). Those who stayed until the end of the study without developing DRC were censored at the end of the study (right censored). To investigate whether recipients’ survival was the same between patients on first-line cART regimen and those on second-line/third-line cART regiment, Kaplan–Meier survival analysis was performed. The average time-to-event was estimated for both the first-line cART and second-line/third-line cART and along with 95% confidence intervals (CIs) computed. The survival function was plotted and checked whether it was the same between patients on first-line cART and those on second-line/third-line cART using the log-rank (Mantel–Cox) Chi square. The significance level was set at p < 0.05.

### Ethics approval and consent to participate

Ethical approval to collect data from HIV clinics was sought and obtained from the University of Botswana Review Board and the ethics committee of the Ministry of Health and Wellness, Botswana. Permission to consult clinic record books and systems was also sought and obtained from the clinic management. As this was a record based study, no consent to participate was required.

### Results

Clinic records of 540 patients were reviewed. Nine patients were excluded as they already had been diagnosed with DRCs before cART initiation. This resulted in total study population of 531 patients to be included in the analysis. Three hundred and fourteen (59.1%) participants received cART at PMH clinic while 217 (40.9%) received cART at Bontleng clinic. Of the 531 patients, 368 (69.3%) were females and 163 (30.7%) were males. The mean (SEM) CD4 cell count at cART initiation and after cART initiation, or at the time of data collection, were 139.6 (5.11) cells/mm^3^ and 536.0 (10.16) cells/mm^3^ respectively. Other characteristics are described in Table 1.

**Table 1:** Characteristics of patients attending Princess Marina Hospital HIV clinic and Bontleng HIV clinic in Botswana (N = 531)

Characteristic	Mean (SEM)	Minimum value	Maximum value
Age (years)	41.4 (8.8)	19.0	82.0
Weight-1 (kg)^a^	60.6 (11.8)	17.5	101.0
Weight-2 (kg)^b^	67.9 (14.5)	32.5	117.7
CD4-1 (cell/mm^3^)^c^	139.6 (5.11)	00.0	889.0
CD4-2 (cell/mm^3^)^d^	536.0 (10.16)	25.0	1441.0

*CD4* cluster of differentiation 4, a glycoprotein found on the surface of immune cells like T helper cells and macrophages, *SEM* standard error of the mean, *kg* kilogram, *mm*^*3*^ millimetre cube

^a^Weight-1 = weight before combination antiretroviral therapy initiation

^b^Weight-2 = weight at the time of data collection (after cART initiation)

^c^CD4-1 = CD4 cell count before cART initiation

^d^CD4-2 = CD4 cell count at the time of data collection (after cART initiation)

There were 318 (59.9%) patients on first-line cART, 209 (39.4%) on second-line cART and 4 (0.8%) on third-line cART. At cART initiation: 408 (76.8%) participants had a CD4 count of ≤ 200 cells/mm^3^ compared to 34 (6.4%) who had a CD4 count of ≤ 200 cells/mm^3^ after initiation of cART, 100 (18.8%) participants had a CD4 count of between 200 and 350 cells/mm^3^ compared to 76 (14.3%) who had a CD4 count of between 200 and 350 cells/mm^3^ after initiation of cART, 23 (4.4%) participants had a CD4 count of ≥ 350 cells/mm^3^ compared to 421 (79.3%) who had a CD4 count of ≥ 350 cells/mm^3^ after initiation of cART McNemar’s test show a significant improvement in CD4 cell count after use of cART compared to before cART initiation (Table 2).

**Table 2:** The average time-to-event among recipients of first-line and second-line/third-line cART from Princess Marina Hospital HIV clinic and Bontleng HIV clinic in Botswana (N = 531)

cART regimen	Mean survival time or average time-to-the event (years)			
	Estimate	Standard error of the mean	95% confidence interval	
			Lower boundary	Upper boundary
First-line	10.6	0.3	10.1	11.1
Second-line/third-line	12.7	0.3	12.2	13.2
Average	11.7	0.2	11.3	12.2

**Fig. 1:**
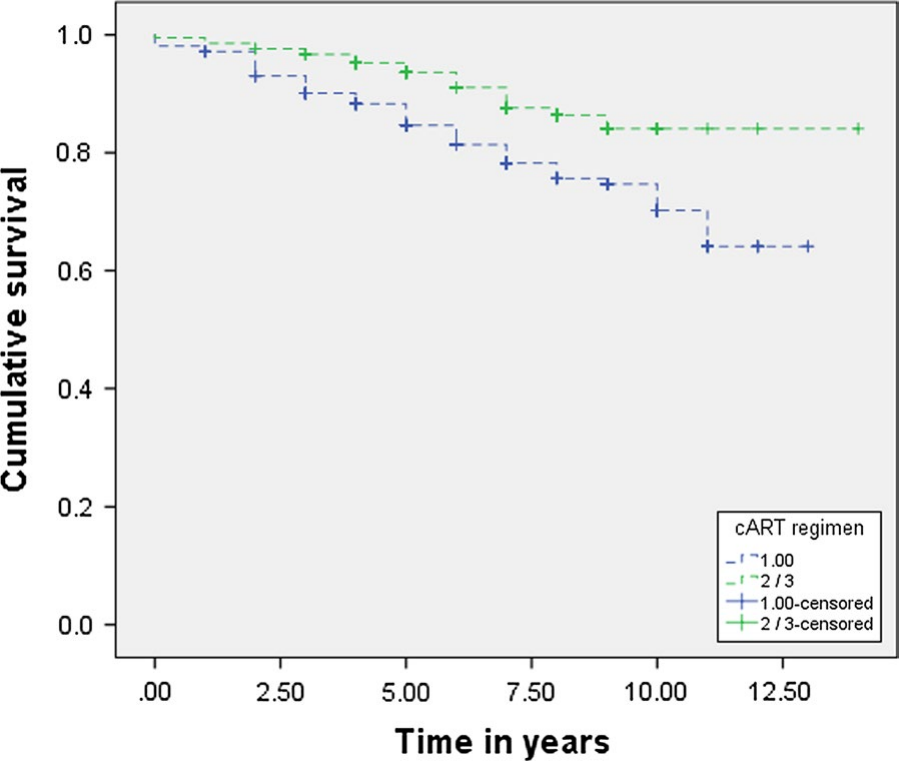
Kaplan–Meier survival curves of recipients of first-line and second-line/third-line cART (N = 531)

Kaplan–Meier survival curves (Fig. 1) demonstrate a significant (p < 0.003) difference between the two exposure variables of interest, first-line cART and second-line/third-line cART

DRC development Log-rank (Mantel–Cox) Chi square = 8.98; df = 1, p = 0.003

Four hundred and forty two (83.2%) patients did not develop any type of DRC and were censored, 89 patients (16.8%) developed various DRCs, namely hypertension (39.6%), lipodystrophy (18.9%), high blood pressure (17.16), overweight (9.0%), renal failure (8.1%), hyperlipidemia (6.3%) or cardiomyopathy (0.9%).

The total time of exposure to cART was 3316 PY, the total number of events or DRCs was 89, corresponding to an incidence density of DRCs of 26.8/1000 PY (95% CI 20.1–32.7). Results from the Kaplan–Meier analysis (Fig. 1) showed of the patients on first-line cART, 252 (79.2%) were censored and 66 (20.8%) had DRC event. Out of those on second-line/third-line cART, 190 (89.2%) were censored and 23 (10.8%) had the events.

Results in Additional file 2: Table S1 show estimates of the average time-to-event (DRC) among recipients of first-line cART (10.6 ± 0.3 years, 95% CI 10.1–11.1) and those of second-line/third-line cART (12.7 ± 0.3 years, 95% CI 12.2–13.2) as well as the average time-to-event for both first-line and second-line/third-line cART. The log-rank (Mantel–Cox) Chi square showed a significant (X^2^ = 8.98, p = 0.003) survival difference between recipients on the first-line cART regimen and those on second-line/third-line cART regimen.

### Discussion

The incidence of DRCs in the study population is relatively high compared to those reported in America, Australia or Europe [5, 18] but was significantly lower than rates reported in some Sub Saharan settings [19]. While cART improves survival, they are also known to cause DRCs among some recipients [11, 20]; depending on the regimen and the duration of exposure, recipients may take longer or shorter time to the development of DRCs [5, 21].

In this study, the second-line/third-line cART regimen had a longer time-to-event, while the first-line cART had a shorter time-to-event. The Kaplan–Meier survival function showed a significant difference in every year of exposure to cART between recipients who were on first-line regimen and those who were on second-line/third-line regimen; suggesting that recipients on first-line cART regimen had a higher risk of developing the outcome before those on second-line/third-line cART regiment. This calls for more research on cART adverse effects in order to identify regimens that minimize these effects while giving to recipients longer survivals to unwanted health events (DRCs).

## Limitations

As a retrospective study, some important data such as family diabetes’ history were missing. In addition, given the drugs were studied in combinations, we were unable to identify which one provided the longest survival to recipients. However, the study has provided some evidence to inform policy and decision-making to improve current care and patient management of PLHIV.

## Additional file

**Additional file 1: Appendix 1.** Standard first- and second-line cART regimens in Botswana.
**Additional file 2: Table S1.** Frequency distribution of diabetes mellitus-related comorbidities among patients attending Princess Marina Hospital HIV clinic and Bontleng HIV clinic in Botswana (N = 89).

## References

[1] Salehian B, Bilas J, Bazargan M, Abbasian M (2005). Prevalence and incidence of diabetes mellitus in HIV- infected minority patients on protease inhibitors. J Natl Med Assoc.

[2] Reid MJ, Mosepele M, Tsima BM, Gross R (2012). Addressing the challenge of the emerging NCD epidemic: lessons learned from Botswana’s response to the HIV epidemic. Public Health Action..

[3] Butt AA, Fultz SL, Kwoh K, Kelley D, Skanderson M, Justice AC (2004). Risk of diabetes mellitus in HIV infected veterans Pre- and Post-HAART and the role of HCV Co-infection. Hepatology.

[4] Ministry of Health (2012). “The Models of Care” Project: an analysis of the national antiretroviral treatment program (MASA), 2007–2011: program effectiveness, cost to the country and clinical effectiveness.

[5] De Wit S, Sabin CA, Weber R, Worm SW, Reiss P, Cazanave C (2008). Incidence and risk factors for new-onset diabetes in HIV-infected patients: the data collection on adverse events of anti-HIV drugs (D:A:D) study. Diabetes Care.

[6] Butt AA, McGinnis K, Rodriguez-Barradas MC, Crystal S, Simberkoff M, Goetz MB (2009). HIV infection and the risk of diabetes mellitus. AIDS..

[7] Brown TT, Li X, Cole SR, Kingsley LA, Palella FJ, Riddler SA (2005). Cumulative exposure to nucleoside analogue reverse transcriptase inhibitors is associated with insulin resistance markers in the multicentre AIDS cohort study. AIDS..

[8] Daar ES, Tierney C, Fischl MA, Sax PE, Mollan K, Budhathoki C (2011). Atazanavir plus ritonavir or efavirenz as part of a 3-drug regimen for initial treatment of HIV-1. Ann Intern Med.

[9] Van der Valk M, Kastelein JJ, Murphy RL, van Leth F, Katlama C, Horban A (2001). Nevirapine-containing antiretroviral therapy in HIV-1 infected patients results in an anti-atherogenic lipid profile. AIDS..

[10] Young F, Critchley JA, Johnstone LK, Unwin NC (2009). A review of co-morbidity between infectious and chronic disease in Sub Saharan Africa: TB and diabetes mellitus, HIV and metabolic syndrome, and the impact of globalization. Global Health..

[11] Samaras K (2009). Prevalence and pathogenesis of diabetes mellitus in HIV-1 infection treated with combined antiretroviral therapy. J Acquir Immune Defic Syndr.

[12] ICD-10-CM (2015). Codebook Index.

[13] Botswana National HIV and AIDS Treatment Guidelines (2012). Ministry of health and wellness report.

[14] Handbook of the Botswana Integrated HIV Clinical Care Guidelines. Ministry of Health and Wellness Report. Gaborone; 2016.

[15] Statistics Botswana (2013). Botswana AIDS Impacts Survey IV, BAIS IV. Gaborone.

[16] Hennekens CH, Buring JE, Mayrent SL (1987). Epidemiology in medicine.

[17] Brown TT, Coles SR, Li X, Kingsley LA, Palella FJ, Ridler SA (2005). Antiretroviral therapy and the prevalence and the incidence of diabetes mellitus in the multicentre AIDS cohort study. Arch Intern Med.

[18] Justman JE, Benninng L, Danoff A, Minkoff H, Levine A, Greenblatt RM (2003). Protease inhibitor use and the incidence of diabetes mellitus in a large cohort of HIV-infected women. J Acquir Immune Defic Syndr.

[19] Okello S, Kanyesigye M, Muyindike WR, Annex BH, Hunt PW, Haneuse S (2015). Incidence and predictors of hypertension in adults with HIV-initiating antiretroviral therapy in south-western Uganda. J Hypertens.

[20] Bisson G, Gross R, Miller V, Weller I, Walker A, Arlett P (2003). Monitoring of long-term toxicities of HIV treatments: an international perspective. AIDS..

[21] Van Oosterhout JJ, Mallewa J, Kaunda S, Chagoma N, Njalale Y, Kampira E, Mukaka M, Heyderman RS (2012). Stavudine toxicity in adult longer-term art patients in Blantyre, Malawi. PLoS ONE.

