# Supplementary material for: Incidence of diabetes mellitus-related comorbidities among patients attending two major HIV clinics in Botswana: a 12-year retrospective cohort study
Source: BMC Res Notes. 2018 Feb 1;11:90. doi: 10.1186/s13104-018-3144-9 (PMC5796438; doi:10.1186/s13104-018-3144-9)
Supplement: Supplementary file 1 — Additional file 1: Appendix 1. Standard first- and second-line cART regimens in Botswana. [file 13104_2018_3144_MOESM1_ESM.docx]

**Appendix**

**Standard First and Second Line ART Regimens in Botswana**

The Botswana National ART programme also called Masa was launched in 2002. Over the years the Ministry of health has issued and updated ARV treatment guidelines based on WHO recommendations. First line and second line regimens have therefore been modified over the years from 2002-2016. Between 2002 and 2015, the period for this study, the treatment regimens were as per table below adapted from the 2012 and before 2016 treatment guidelines:

**Standard First and Second Line ART Regimens in Botswana [Reference No 14 and 15]**

| **First line** | **First line Modifications** | **Second line** | **Second line Modifications** |
| --- | --- | --- | --- |
| AZT +3TC (CBV-combivir) + EFV  AZT + 3TC +NVP  AZT +DDI+ EFV  AZT+ DDI +NVP | **TDF renal toxicity w/o CVD risk:**  ABC/3TC/DTG  (If CVD rash: Consult HIV specialist)  **CNS Toxicity and/or**  **Hepatic Toxicity:**  TRU/DTG | TDF+FTC +ALU | AZT Anemia and/or  TDF Renal Toxicity: ABC/3TC/DTG |
| TDF+ FTC (or 3TC) + EFV  TDF+ FTC (or 3TC)+ NVP |  | CBV + ALU | If anemic ABC+ 3TC+ ALU |
| D4T + 3TC +EFV  D4T + 3TC +NVP  DDI +3TC +EFV  DDI+ 3TC +NVP |  | TDF+FTC=ALU | If renal insufficiency but no anemia: CBV+ALU  If renal insufficiency and anemia: ABC + 3TC+ALU |

**The third-line regimen** was made up of other alternative combination or salvage therapy. This was deployed in case of failure of both the standard first and second-line regimens.
