# Supplementary material for: Incidence of diabetes mellitus-related comorbidities among patients attending two major HIV clinics in Botswana: a 12-year retrospective cohort study
Source: BMC Res Notes. 2018 Feb 1;11:90. doi: 10.1186/s13104-018-3144-9 (PMC5796438; doi:10.1186/s13104-018-3144-9)
Supplement: Supplementary file 2 — Additional file 2: Table S1. Frequency distribution of diabetes mellitus-related comorbidities among patients attending Princess Marina Hospital HIV clinic and Bontleng HIV clinic in Botswana (N = 89). [file 13104_2018_3144_MOESM2_ESM.docx]

**Table S1 Frequency distribution of diabetes mellitus-related comorbidities among patients attending Princess Marina Hospital HIV clinic and Bontleng HIV clinic in Botswana (N = 89)**

| **Rank** | **Type of DRC** | **Number** | **Proportion (%)** |
| --- | --- | --- | --- |
| 1 | Hypertension | 44 | 39.6 |
| 2 | Lipodystrophy | 21 | 18.9 |
| 3 | High blood pressure | 19 | 17.1 |
| 4 | Overweight (clinical) | 10 | 9.0 |
| 5 | Renal failure | 9 | 8.1 |
| 6 | Hypercholestrolaemia | 7 | 6.3 |
| 7 | Cardiomyopathy | 1 | 0.9 |
|  | Total | 111* | 100 |

*Some patients had multiple DRCs as illustrated in Figure 1
